# Host Factors That Control Mosquito-Borne Viral Infections in Humans and Their Vector

**DOI:** 10.3390/v13050748

**Published:** 2021-04-24

**Authors:** Chasity E. Trammell, Alan G. Goodman

**Affiliations:** 1 School of Molecular Biosciences, College of Veterinary Medicine, Washington State University, Pullman, WA 99163, USA; chasity.trammell@wsu.edu; 2 NIH Protein Biotechnology Training Program, Washington State University, Pullman, WA 99164-6240, USA; 3 Paul G. Allen School for Global Health, College of Veterinary Medicine, Washington State University, Pullman, WA 99164, USA

**Keywords:** innate immunity, mosquitoes, arboviruses

## Abstract

Mosquito-borne viral infections are responsible for a significant degree of morbidity and mortality across the globe due to the severe diseases these infections cause, and they continue to increase each year. These viruses are dependent on the mosquito vector as the primary means of transmission to new vertebrate hosts including avian, livestock, and human populations. Due to the dynamic host environments that mosquito-borne viruses pass through as they are transmitted between vector and vertebrate hosts, there are various host factors that control the response to infection over the course of the pathogen’s life cycle. In this review, we discuss these host factors that are present in either vector or vertebrate models during infection, how they vary or are conserved between hosts, and their implications in future research pertaining to disease prevention and treatment.

## 1. Introduction

Vector-borne diseases pose a significant global health burden. Global climate changes have enabled various arthropod vectors to expand into previously uninhabitable regions which increases potential exposure to at-risk populations [1,2]. In particular, mosquito populations have expanded, and this has resulted in increasing occurrence of mosquito-borne disease [3,4], notably viruses such as West Nile virus (WNV), dengue virus (DENV), Zika virus (ZIKV), and chikungunya virus (CHIKV) [5]. While mosquitoes are also responsible for transmission of hazardous parasitic diseases such as plasmodia and filariasis parasites, their role as transmitters of viral pathogens is of particular interest to epidemiologists and infectious disease monitoring groups. Recent reports by the World Health Organization (WHO) have indicated a steady or downward trend of global malaria cases [6], while viral cases have appeared to increase in both frequency and severity [5,7,8]. This has resulted in the WHO to designate DENV and related mosquito-borne viruses as significant concerns that need to be addressed at the global level in 2020 [9].

Mosquito-borne viruses utilize the mosquito as a natural reservoir for replication and vector for transmission into vertebrate populations. Depending on the virus, transmission can occur in both sylvatic and urban settings during the bloodmeal between mosquito and vertebrate hosts such as primates, birds, and humans [10,11]. Once infected, some vertebrate hosts are unable to clear acute infection and can develop viremia which results in infectious virions circulating in the blood and lymphatic fluid. This would, in turn, result in potential virus transmission from vertebrate to mosquito in subsequent bloodmeals [10]. The transmission cycle between mosquito and vertebrate populations requires constant monitoring to identify new infections in each host type as this would indicate active viral transmission within a community or region.

Viruses that undergo this transmission cycle encounter various environmental and host factors as virions move between mosquito and vertebrate hosts. Immune responses to mosquito-borne viruses vary between vector and vertebrate hosts as the sophistication and complexity in the cellular environment vary between species. This does not, however, mean that there are no evolutionarily conserved responses that are shared between organisms. In fact, at the level of innate immunity, there are many shared antiviral mechanisms that are conserved between species. In the following review, we provide a summary of conserved host factors responsible for initiating antiviral responses against mosquito-borne viruses in mosquito and human systems, how these factors vary between organisms, and how these responses provide a foundational understanding for future vector control and therapeutic research.

## 2. Mosquito Antiviral Immunity

Mosquito-borne viruses utilize multiple mosquito species as the primary means of transmission into higher-level vertebrate species such as birds and mammals. While these viruses can be transmitted within a vertebrate population by routes such as blood transfusion [12,13], sexual transmission [14], or in utero transmission [15], the mosquito is the predominate means of transmission and is responsible for a majority of disease cases within a community. Mosquitoes acquire a viral infection during the bloodmeal exchange from an infected vertebrate, but there is evidence suggesting vertical transmission of certain viruses such as ZIKV from female mosquitoes to eggs [16,17]. Once the virus-containing bloodmeal is ingested and digested in the midgut, the virus escapes the midgut and systemically infects distal tissues including the ovaries, fat body, and salivary glands [18]. As viremia is reached in the mosquito, the saliva becomes infected with high levels of infectious virions that induces activation of chemosensory-related genes that affects feeding behavior [19]. Once the salivary glands become infected, the mosquito becomes a competent vector for future virus transmission during subsequent bloodmeals. Mosquitoes can be co-infected with different viruses simultaneously without compromising vector competence or survival, such as *Aedes aegypti* infected with DENV, ZIKV, and CHIKV; the mosquito can then transmit these viruses simultaneously [20,21].

From the initial digestion of the virus-containing bloodmeal to transmission, the mosquito initiates different cellular responses to control the virus without affecting host survival. Physical tissue barriers are present along the mosquito digestive tract, and the virus must pass through this barrier in order to become systemic. Then, the virus reaches the salivary glands and ovaries for horizontal and vertical transmission, respectively. Broad and specific antiviral immune signaling responses are also crucial to reduce a virus’s ability to establish itself within the mosquito. The antiviral barriers involved in protecting mosquitoes from mortality due to viral infection are generally well conserved across genera such as *Culex* and *Aedes*.

### 2.1. Innate Immune Responses in the Mosquito

The mosquito, like other invertebrate species, lacks the canonical adaptive immune system found in more complex vertebrate species such as humans [10]. Instead, mosquitoes utilize response pathways that are heavily conserved across metazoa and are critical in the insect immune system and innate immunity. As opposed to the development of immunological memory via the adaptive immune system, the innate immune system involves recognition of pathogen-associated molecular patterns (PAMPs) by pattern recognition receptors (PRRs) that activate immune signaling pathways which ultimately: (1) induces transcription of downstream antiviral effector proteins, or (2) recruits immune cells that can initiate cellular and humoral signaling to localized infection. The primary focus of innate immune signaling is to initiate an immediate response to infection that aims to clear and keep the virus localized. The innate immune system has been heavily dissected and studied in the context of mammalian systems, the *Drosophila melanogaster* model organisms, and mosquito species to varying degrees. Taken together, the role and significance of innate immune signaling against mosquito-borne viruses are a well-established and continuously growing field of study.

**Figure 1:**
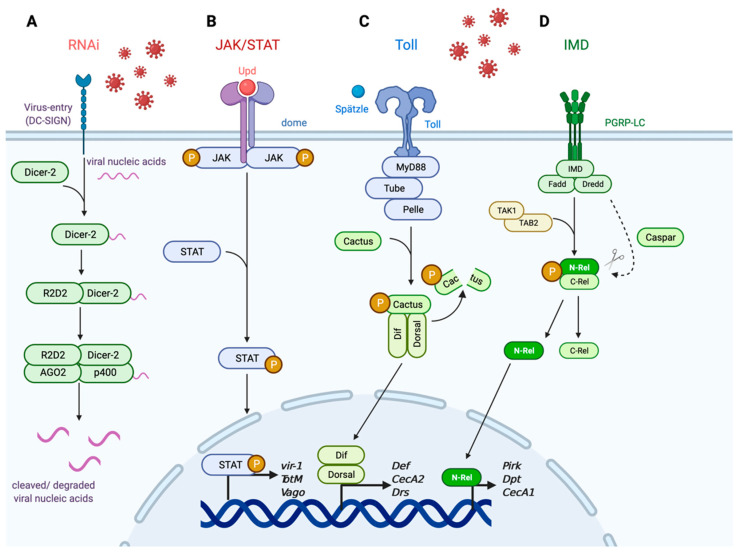
Innate immune signaling in insect and mosquito systems. The innate immune system is heavily conserved across species and involves various signaling pathways induced in response to viral nucleic acids (RNAi) or detection of upstream antiviral effectors (JAK/STAT, Toll, IMD). (**A**) The RNA interference (RNAi) pathway functions to detect cytosolic dsRNA or DNA that is indicative of viral infection. (**B**) The JAK/STAT pathway, which is conserved between mosquito and human species, induces transcription of downstream antiviral effector genes. (**C**) The Toll pathway functions to respond to Gram-positive bacterial and fungal infection and is present in the form of TLR in mammals. (**D**) The IMD pathway is activated during Gram-negative bacterial infection and is similar to the TNF/NF-kB pathway. Adapted from “Blank Pathway (Linear)”, by BioRender.com (2021). Retrieved from https://app.biorender.com/biorender-templates [42].

One of the more significant defense responses against a multitude of viral infections in the mosquito is the RNA interference (RNAi) pathway (Figure 1A). RNAi signaling is a heavily conserved pathway across invertebrate and vertebrate organisms and is involved in regulating gene expression [22]. In invertebrate organisms, the RNAi pathway also functions as an antiviral response pathway that is activated by nucleic acids that are the by-products of the replicative process during viral infection [23,24,25]. Specifically, viral nucleic acids such as double-stranded RNA (dsRNA) are detected by the endoribonuclease Dicer-2 which binds to and cleaves the larger dsRNA into smaller RNA duplexes [26,27]. R2D2 then binds to the small RNA duplexes, and the protein–viral nucleic acid complex is loaded into the RNA-induced silencing complex (RISC) [28]. Once associated with RISC, the effector proteins AGO2 and p400 bind to specific sequences within the viral mRNA target to cleave bound viral nucleic acids [29,30,31]. The purpose of this degradation of viral nucleic acids is to clear the intracellular virus before viral replication proteins can hijack the host translational machinery and generate more infectious virion copies. The importance of this pathway has been demonstrated against flaviviruses such as DENV2 and WNV in *Aedes* and *Culex* mosquitoes, respectively [32,33,34], and ZIKV and WNV in *D. melanogaster* [35,36]. RNAi is also important in controlling viral replication and mosquito survival against alphaviruses such as Sindbis virus (SINV) [37] and Semliki Forest virus (SFV) [38]. While RNAi is present in higher-level organisms such as humans, the extent and importance of its role as an antiviral immune regulator remain unclear and it is not yet defined as an antiviral mechanism in mosquito-borne viral infections [39,40,41].

Another important signaling response involved in mosquito antiviral immunity is the JAK/STAT pathway (Figure 1B). Similar to RNAi, JAK/STAT is linked to various host processes beyond immunity including cellular division, maintenance, and regeneration [43,44], along with regulating oogenesis in insect species [45,46]. Unlike the RNAi pathway, however, the functional role of JAK/STAT as a cellular and antiviral regulator is well conserved between vertebrate and invertebrate hosts [47,48] and is associated with responding to various viral infections including WNV [49], ZIKV [50,51], and DENV [52,53,54]. JAK/STAT is an effector response pathway that activates an intracellular signaling cascade and induces downstream antiviral genes in response to infection [55]. Upon infection, the extracellular ligand unpaired (Upd) is secreted [56,57] and binds to the receptor domeless (dome) expressed on neighboring hemocytes or related immune cells [58]. Binding results in the activation of the intracellular Janus kinase hopscotch (hop) [59] and phosphorylation of transcription factor STAT [60,61]. Phosphorylation then induces the dimerization and nuclear import of STAT to promote transcription of downstream antiviral effectors such as *vir-1* [55] and *TotM* [56]. Vago is a secreted cytokine that activates JAK/STAT signaling in *Cx. quinqufasciatus*, and *Vago* is induced in a Dicer-2-dependent manner [62]. JAK/STAT signaling is conserved between invertebrate and vertebrate species as well as its functional role in an innate immune response to viral infections. In humans, JAK/STAT signaling is partially involved in the generation of type-I interferon (IFN)-stimulated responses and has been shown to be active in the presence of ZIKV [63], WNV [64], and Japanese encephalitis virus (JEV) [65]. Since Vago restricts WNV in *Cx. quinqufasciatus*, its role as an antiviral secreted cytokine is similar to that of mammalian IFN [62].

The Toll and IMD pathways, while primarily associated with antibacterial and antifungal immunity, have also been implicated in antiviral protection within the mosquito and insect models (Figure 1C,D) [66,67]. The Toll pathway is important for defense against Gram-positive bacterial and fungal infections [68,69], whereas IMD is important against Gram-negative bacteria [70,71]. The mechanistic events involved in Toll and IMD signaling have been heavily dissected using the *D. melanogaster* model, and the pathways are evolutionarily conserved in *Aedes* and *Culex* mosquitoes. Toll signaling is initiated when the ligand Spätzle binds to the Toll receptor to activate adaptor proteins MyD88, Tube, and kinase Pelle to induce a phosphorylation cascade that activates degradation of the regulatory factor Cactus following its phosphorylation [72,73,74]. Once Cactus is degraded, the transcription factors Dif and Dorsal are able to translocate into the nucleus to induce transcription of downstream antimicrobial peptides (AMPs) and response genes [75]. The IMD pathway is activated by DAP-type peptidoglycans recognized by peptidoglycan recognition proteins (PGRP-LC) which act as transmembrane receptors that induce a series of phosphorylation and cleavage events in the cytosol. These intracellular events ultimately result in induction of downstream AMPs and response genes [76]. Binding to PGRP-LC induces the formation of a signaling complex composed of the proteins IMD, Fadd, and Dredd [70,77,78]. Dredd cleaves IMD which recruits the Tab2/Tak1 protein complex that induces the phosphorylation and cleavage of the transcription factor Relish (Rel) [79,80,81]. Caspar acts as a negative regulator of IMD by targeting Dredd-mediated cleavage [82]. Cleavage of Rel results in the nuclear translocation of the N-terminus of Rel to induce transcription of effector genes responsible for regulating AMPs and other immune response elements [78,83]. The Toll and IMD pathways have been heavily dissected in the context of antibacterial and antifungal immunity but have also been linked to humoral and cellular antiviral responses in the insect system. For example, *Ae. aegypti* knocked down for *Cactus* and *Caspar* by RNAi exhibited increased Toll and IMD immune signaling during DENV infection and reduced viral replication [84,85]. Toll and JAK/STAT signaling has also been shown to be induced during ZIKV infection in *Ae. aegypti* [51]. In addition, WNV infection in *Culex pipiens* induces Toll signaling in addition to the canonical RNAi and JAK/STAT pathways [86]. It is also important to note that each pathway is involved in hemocytes’ functional role as circulating immune cells against insect-specific [55,87,88,89] and vector-borne viruses such as DENV and ZIKV [51,52,84,86,90]. Finally, Toll and IMD signaling is conserved in the human immune system as the Toll-like receptor (TLR) signaling pathway [91] and NF-kB/TNF signaling pathways [92], respectively. Both TLRs and NF-kB/TNF signaling pathways have been linked as critical defense mechanisms against various RNA viruses including WNV [93,94,95,96].

### 2.2. Physical Barriers in the Mosquito

Mosquitoes primarily become infected through ingestion of a bloodmeal containing a virus. Due to this infection route, virions undergo various environmental pressures and conditions as they move from the midgut to distal tissues such as the salivary glands and ovaries which are involved in horizontal and vertical transmission, respectively. The physical barriers that the viruses overcome in order to reach viremia conditions are a significant component in the mosquito’s response to infection. Comparatively, the physical barriers involved in the mosquitoes’ and humans’ response in infection vary significantly as the different organ systems and cellular pressures encountered would utilize different host factors to respond to viral infection.

Perhaps the most significant tissue functioning as a physical barrier in preventing viremia in the mosquito is the midgut, the organ responsible for the digestion of an ingested bloodmeal and absorbance of essential nutrients [97]. The virus must first overcome the midgut infection barrier, which is when the virus moves from the gut lumen to the midgut epithelial cells. Upon infecting and replicating in the midgut epithelial cells, the virus then passes the midgut escape barrier and basal lamina to disseminate into the hemocoel [97,98]. Once the virus breaches the midgut, virions enter the hemocoel and induce activation of humoral immune responses such as melanization [99] to limit dissemination [100]. Failure to keep infection localized results in viremia that systemically infects distal tissues such as the fat body, hemocytes, and salivary glands [19,101,102]. It is at this point when the virus reaches, modulates signaling events, and establishes itself in the salivary glands that the mosquito becomes a competent vector for virus transmission in future bloodmeals. The dissemination rate into midgut epithelial cells varies between virus and mosquito species as JEV disseminates faster in *Culex* mosquitoes when compared to DENV2 dissemination in *Aedes* [103,104]. Dissemination rates are also enhanced based on the frequency of subsequent bloodmeals, regardless of whether they are infected or not, due to the digestive impact on the midgut integrity and permeability [16,105,106].

For a virus to reach the blood–lymphatic system and become systemic, virions must overcome the physical and chemical barriers of skin tissue, in addition to avoiding immune cells. These differences in how viremia is achieved in the vector and mammalian hosts pose as a potential target for vector control intervention. The different cellular environments that the virus is exposed to during dissemination in the mosquito and human hosts implies that targeted therapeutics would have varying degrees of success. For example, recent studies have demonstrated that targeting signaling events involved in digestive and nutritional acquisition may prime mosquitoes for viral infection and reduce viral replication and the likelihood of transmission to subsequent hosts [49,107,108,109]. This is evident in the implementation of using *Wolbachia*, an endosymbiont present in various insect and arthropod species, as a means of vector control due to its established effect on reducing vector competence and viral replication in mosquito populations [52,110,111]. Additionally, stimulating the insulin/IGF-1 signaling pathway has been shown to reduce infection in mosquito vectors [49,107,108]. While targeting nutritional and digestive events may be effective in limiting viral activity in the mosquito, targeting similar processes in humans or other vertebrates may not be as effective as the virus would not undergo the same cellular pressures. In the case of insulin/ IGF-1 signaling, in which insulin has a broad effect on transcriptional activity beyond immune signaling, it may be possible to implement similar insulin-dependent strategies for both by targeting different insulin targeted-downstream host factors [109,112,113,114].

### 2.3. Variability between Mosquito Species and Viruses

Specific virus transmissions are generally linked to certain mosquito genera or species. For example, *Aedes* mosquitoes are primarily associated with hemorrhagic- or arthritic-inducing viruses such as DENV, CHIKV, and yellow fever virus (YFV) transmission, whereas *Culex* mosquitoes are associated with encephalitic viruses such as WNV, JEV, and St. Louis encephalitis virus (SLEV). Each genus’s geographic range does overlap to a certain degree with significant overlap within the Northern and Southern tropics, but each genus does possess a certain unique range as habitation becomes more polar within Africa and Southeast Asia [3,4,115,116]. There is also evidence of specific genus activity within the Northern and Southern tropics based on present environmental pressures such as elevation, population density, and available nutritional sources [2,117]. This correlates to disease incidence within these areas as well as expansion of mosquito populations within the regions [7,118].

Current studies have primarily focused on how specific mosquito species respond to viral infection without comparison to how other species may respond to the same pathogen. ZIKV and Rift Valley fever virus (RVFV), for example, are able to infect both *Aedes* and *Culex* mosquitos but to differing levels of success depending on the species and virus strain [119,120,121]. Research thus far as indicated that certain immune signaling and physical barriers play an important role in antiviral responses, but there is limited understanding as to how multiple canonical or novel signaling pathways may interact with one another to achieve the most effective immune response. While there is still much to discover regarding how different mosquito populations respond to and regulate the multitude of arboviruses that pose a threat to human populations, new studies are beginning to compare the related and unique host factors within mosquito populations and how they may lead to either broad or mosquito-specific intervention targets.

## 3. Human Antiviral Immunity

Mosquito-borne viruses pose a global health threat as they can be transmitted to humans with limited therapeutics or preventatives available. Unlike the mosquito vector, vertebrate hosts have evolved to possess two forms of immunity: the innate immune response, which is heavily conserved across species as previously discussed, and the adaptive immune response. The latter form of immunity is responsible for specific and long-lasting immunological memory associated with humoral and cellular immune responses. In the context of immune responses during an active viral infection, both branches of host immunity are involved and impact disease morbidity, mortality, and long-term immunity. Many of the host factors and signaling pathways present in the mosquito are also conserved in humans (Table 1). Specifically, the innate immune response pathways previously discussed are present to some orthologous or functional degree. Variability between mosquito and human host factors active during viral infections primarily exists in the form of physical defense barriers and adaptive immune responses.

### 3.1. Transmission and Physical Barriers in Human Hosts

**Figure 2:**
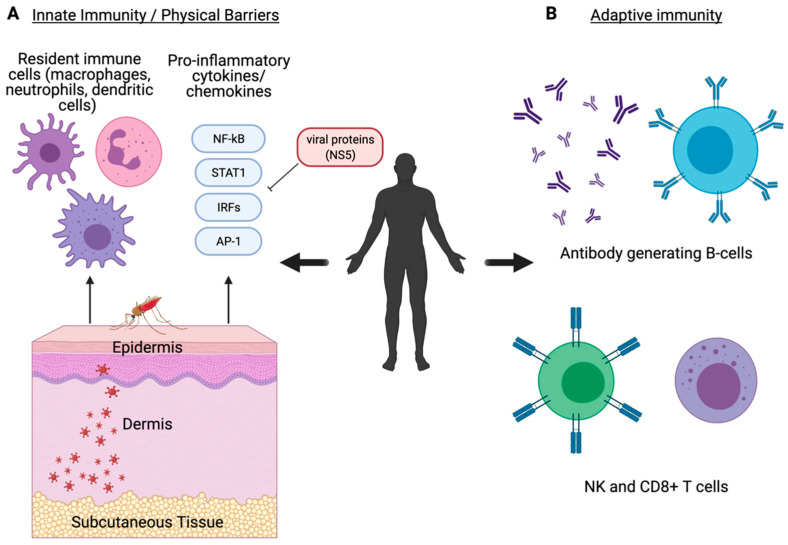
Innate and adaptive immune signaling in the human system. Humans defend against viral infections using physical barriers, innate immune signaling, and adaptive immune signaling responses. (**A**) Innate immune responses occur at physical barriers and involve the secretion of pro-inflammatory cytokines and chemokines by resident immune cells such as macrophages, neutrophils, and dendritic cells [126,127,128,129,131,132,133]. Components of the innate immune system are well conserved in both mosquitoes and humans. (**B**) The adaptive immune response primarily involves the generation of specialized and specific antibody-producing B cells, NK cells, and CD8+ T cells [134,135,136,137,138,139,140]. The adaptive immune response is a more evolved form of immunity that is unique to vertebrate organisms. Created with BioRender.com [42].

The human host initiates an immediate immune response following a mosquito bloodmeal in which the virus-infected saliva is ejected from the mosquito hypopharynx into the skin epidermis. The saliva contains various host-derived salivary factors that can enhance viral transmission and reduce pro-inflammatory responses initiated by the human host [122,123,124]. This includes various mosquito-derived factors including anticoagulants [125] and deregulators that disrupt recruitment of immune cells such as macrophages and neutrophils [126,127,128,129]. Ultimately, these salivary factors result in enhanced cell infection and viral replication that assists in viral dissemination [130]. Virions infect local resident cell populations including keratinocytes [131], dermal dendritic cells (DCs), and Langerhans cells (Figure 2A) [132]. DCs are responsible for movement of a virus into the local lymph node where replication and viremia are induced [132]. Permissive tissues typically vary between virus type but include the spleen (DENV, RVFV), liver (YFV, WNV), and neuronal tissue (ZIKV, JEV), amongst others. This tissue tropism coincides with the disease manifestation caused by each virus.

### 3.2. Innate and Adaptive Immune Responses in the Human Host

Innate immune responses to viral infection in the human host involve both conserved responses present in the mosquito as well as more effective, specific responses evolved in vertebrate organisms. Specifically, the innate immune signaling pathways previously discussed within the mosquito are functionally present in humans in the forms of more evolutionarily advanced responses that can act both independently and cross-talk with other pathways (Table 1). Local immune cells induce expression of pro-inflammatory cytokines and type I interferon (IFN) responses including IFN-α and IFN-β [133]. Induction of IFN and downstream antiviral response genes are regulated by PRRs such as the RIG-I-like receptors (RLR), TLRs, and cGAS/STING that regulate transcription factors such as NF-kB and IRF3, which then stimulates JAK/STAT signaling to induce IFN-stimulated genes (ISG) [141]. This cytokine-mediated signaling indicates the presence of active infection and results in the recruitment of immune cells (Figure 2A) [64,65]. Proper function of these response elements is critical for host survival [142,143]. The NS5 protein expressed by WNV [144], ZIKV [145], DENV [146], JEV [65], and YFV [147] has shown to mediate host immune evasion as an antagonist to IFN signaling which results in enhanced viral replication (Figure 2A). CHIKV NS2 also functions as an antagonist of IFN signaling by inhibiting activation of JAK/STAT [148].

In addition to the innate immune signaling conserved across species, vertebrate organisms have an evolved adaptive immune system to more effectively respond to infection. Components of the adaptive immune system make up the foundation of what is referred to as immune memory by developing established B and T cell populations that can more effectively and specifically recognize, neutralize, and degrade infectious virions during and in subsequent infections. DCs and macrophages act as antigen-presenting cells and link innate and adaptive immune responses to induce expression of pro-inflammatory cytokines and chemokines and recruitment of cell populations involved in the cell-mediated response including NK cells [134,135] and neutrophils [127,136]. As infection progresses, IgG-secretory B cells and CD8+ T cell populations develop to effectively neutralize and inhibit further viral replication (Figure 2B) [137,138,139,140]. While the host factors involved in innate immune signaling are conserved between mosquitoes and humans, the adaptive immune response has evolved its own unique subset of host factors to enhance host immunity against viral pathogens (Table 1).

### 3.3. Impacts on Morbidity and Mortality

Following a successful immune response, humans are typically able to clear viral infection and generate some protective immunity for potential future infections. There is evidence, however, indicating that even upon clearing an active infection, certain host factors can greatly influence the outcome of future infections against the same or related mosquito-borne viruses. Perhaps the most significant example is the phenomenon known as antibody-dependent enhancement (ADE). ADE is a result of a prior infection generating antibodies that, upon subsequent infections with a similar or related virus, enhances viral entry, replication, and the likelihood of severe disease [149]. This permits partially bound virion–antibody complexes to bind to Fc receptors present on immune cells to mediate increased entry, mass virion replication, and premature release of virions [150,151,152]. DENV is the best example of mosquito-borne virus ADE as subsequent infection with a different serotype [152] or ZIKV [153] may result in this increased viral uptake in immune cells and an increased likelihood of presenting symptoms associated with disease such as hemorrhagic fever or neurological damage. This, in turn, is followed by greater disease severity and risk of mortality [154,155,156]. ADE is a significant concern in the production and implementation of DENV vaccines and as such has caused delays in their effective development and implementation to the general public [157].

Dysfunctional insulin signaling in humans is linked to impaired immunity to mosquito-borne viruses. Diabetic individuals are more prone to developing severe disease symptoms and mortality against ZIKV [158], WNV [159,160], and DENV [161]. Previously medical professionals thought this was due to an overall reduction in host signaling and regulatory processes, but now studies have begun to identify that such viruses impact and target components of insulin signaling to cause disease pathology. The NS4A and NS4B proteins expressed on ZIKV reduce AKT-mTOR signaling, which is targeted in insulin treatment and causes destruction of neuronal tissues that is a hallmark of disease [114]. While the effect that insulin has on human immunity during arboviral infection is still a relatively unexplored field, it may be an ideal candidate for future disease intervention that could be applied at the vector level as well.

Co-infection with multiple arboviruses poses different effects between human and mosquito hosts. While humans may present with more severe clinical symptoms or competing viral replication [162], mosquitoes co-infected with different viruses experience little obvious hazardous phenotypes [20,21] and may even enhance viral replication and the likelihood of transmission [163]. Whether this is due to variability in host factors or viral replication mechanisms is still under investigation, but this phenomenon does present another example in which mosquito and human immune responses to arboviruses vary at the molecular level due to available host factors.

## 4. Outlook

As evident in the gradual increase in the number and severity of clinical cases as well as the expansion of mosquito activity across the globe, the looming threat that mosquito-borne viruses pose is of significant concern and must be addressed at both the vector and clinical levels. Understanding the host immune responses and how they are varied between organisms is an important step in identifying more effective targets for vector control and therapeutics. More importantly, understanding how the responses are similar between mosquito-borne viruses is of great value as it permits research in broad or virus-specific targeting. As summarized in Table 1, the host factors involved during viral infection in mosquitoes and humans are well conserved with some variability regarding host physical barriers and adaptive immunity.

Further investigation is required into identifying and evaluating the importance of certain antiviral immune responses in both humans and mosquitoes. For example, vector-control mechanisms such as the endosymbiont *Wolbachia*, while reducing viral load in mosquitoes infected with ZIKV and DENV [110,111], may be pro-viral for other related viruses such as WNV [164]. This indicates that there may be virus-specific variations among related pathogens that result in potential, broad antiviral preventatives being less effective. This is also the case in humans as responses that are important against one virus may be insignificant or detrimental for another [157]. One example of a potential cross-species antiviral target, as previously discussed, is how insulin/IGF-1 signaling regulates mosquito and human immunity. Since this pathway possesses a broad effect on homeostatic activity in both organisms, insulin-mediated immunity may be achieved by targeting different downstream host factors or pathways. While studies into insulin-mediated arboviral immunity are not well established in humans, there is an established effect of dysfunctional insulin signaling on patient survival and virus activity for ZIKV and WNV [114,160]. Research in and implementation of more effective antivirals in both mosquitoes and humans are necessary and require a greater understanding regarding the conserved and differing host factors that respond to these zoonotic infections.

## Figures and Tables

**Table 1:** Host factors involved in responses to mosquito-borne viruses.

		RNA Interference	JAK/STAT	Toll	IMD/TNF	Physical Barriers	Adaptive Immunity
	
Mosquitoes		Dicer-2 AGO2 Drosha R2D2 p400 piwi4 *Ppo8*	Hop STAT1 *Vago* *vir-1*	Toll dMyD88 Dorsal DIF Cactus *Spätzle*	Relish Caspar Fadd Dredd	Hemocytes *Midgut epithelium*	N/A
Humans		Dicer AGO Drosha TARBP2 Piwi	JAK STAT1/2 *RIG-I* *MDA5* *IFN*-α/β	TLRs MyD88 NF-kB *IFN*-α/β *888IRF7*	NF-kB FAF1 Caspase8/10	Blood–brain barrier *Epidermis* *Dermis*	*B cells* *T cells*

Italicized factors are unique to the specified host or no ortholog exists in the other host.

## Data Availability

Data sharing not applicable.
